# Type I neuregulin1α is a novel local mediator to suppress hepatic gluconeogenesis in mice

**DOI:** 10.1038/srep42959

**Published:** 2017-02-20

**Authors:** Takatomo Arai, Yumika Ono, Yujiro Arimura, Keimon Sayama, Tomohiro Suzuki, Satoko Shinjo, Mai Kanai, Shin-ichi Abe, Kentaro Semba, Nobuhito Goda

**Affiliations:** 1 Department of Life Science and Medical Bioscience, School of Advanced Science and Engineering, Waseda University, Tokyo, 162-8480, Japan; 2 Center for General Education, Kumamoto Health Science University, Kumamoto, 861-5598, Japan

## Abstract

Neuregulin1 is an epidermal growth factor (EGF)-like domain-containing protein that has multiple isoforms and functions as a local mediator in the control of various cellular functions. Here we show that type I isoform of neuregulin1 with an α-type EGF-like domain (*Nrg1α*) is the major isoform in mouse liver and regulates hepatic glucose production. Forced expression of *Nrg1α* in mouse liver enhanced systemic glucose disposal and decreased hepatic glucose production with reduced fasting blood glucose levels. Nuclear forkhead box protein O1 (FoxO1) and its downstream targets, PEPCK and G6Pase, were suppressed in liver and isolated hepatocytes by *Nrg1α* overexpression. In contrast, silencing of *Nrg1α* enhanced glucose production with increased PEPCK and G6Pase expressions in cAMP/dexamethasone-stimulated hepatocytes. Mechanistically, the recombinant α-type EGF-like domain of NRG1α (rNRG1α) stimulated the ERBB3 signalling pathway in hepatocytes, resulting in decreased nuclear FoxO1 accumulation via activation of both the AKT and ERK pathways. In addition, acute treatment with rNRG1α also suppressed elevation of blood glucose levels after both glucose and pyruvate challenge. Although a liver-specific deletion of *Nrg1* gene in mice showed little effect on systemic glucose metabolism, these results suggest that NRG1α have a novel regulatory function in hepatic gluconeogenesis by regulating the ERBB3-AKT/ERK-FoxO1 cascade.

*De novo* synthesis of glucose from non-carbohydrate substrates, such as lactate, glycerol, and alanine, in the liver, a process known as hepatic gluconeogenesis, is essential for maintaining blood glucose levels in a narrow range during starvation. The brain uses glucose as a favourable energy source even under low blood glucose levels; thus, sustained hepatic glucose production is required to circumvent life-threatening hypoglycaemia in fasting conditions. Aberrant activation of liver gluconeogenesis, in contrast, is strongly associated with hepatic insulin resistance and leads to abnormal increases in both fasting and postprandial blood glucose levels in diabetes mellitus. The coordinated regulatory mechanisms of hepatic glucose production have been extensively studied, and key hormones, such as insulin, glucagon, and glucocorticoids, have been shown to serve as crucial modulators in liver gluconeogenesis. In addition to these classical hormones, many extrahepatic organ-derived molecules, known as adipokines and myokines, have recently been reported to play important roles in the regulation of hepatic glucose production^1^,^2^. The liver also participates in metabolic regulation by secreting several humoral factors, such as fetuin-A, fibroblast growth factor 21, and selenoprotein P, which modulate systemic glucose tolerance and insulin sensitivity without any apparent effects on liver gluconeogenesis^3^,^4^. However, the importance of locally released proteins from the liver in hepatic glucose production is unclear.

Neuregulin1 (NRG1) is a growth factor that contains an epidermal growth factor (EGF)-like domain and consists of six major types with differing N-terminal regions. The various isoforms are generated due to either alternative splicing or use of 5′ flanking regulatory regions in humans^5^. NRG1 is actively cleaved at the juxtamembrane region by type I transmembrane proteases, such as β-site amyloid precursor protein cleaving enzyme, releasing the extracellular portion of NRG1. This extracellular molecule contains a biologically active EGF-like domain and functions as a local mediator in either an autocrine or paracrine fashion^6^. The cleaved ectodomain of NRG1 serves as a ligand for two ERBB receptor tyrosine kinases, ERBB3 and ERBB4^5^. Consequently, it activates intracellular signalling cascades to regulate diverse biological processes involved in heart development, cancer, and schizophrenia^7^,^8^,^9^. NRG1 also stimulates glucose uptake by translocating the glucose transporter and increases insulin sensitivity in skeletal muscles^10^,^11^, indicating that it is regulator of glucose metabolism. This hypothesis is further supported by recent reports showing that a single injection of the recombinant β-type EGF-like domain of NRG1 enhances systemic glucose disposal after glucose challenge by activating ERBB3-mediated AKT phosphorylation in rodents^12^,^13^. In addition, NRG1β2 also shows the same blood glucose-lowering effects by stimulating ERBB3-AKT-FoxO1 signalling cascade and subsequently inhibiting hepatic gluconeogenesis in diabetic mice^14^. These observations indicate that NRG1 has important roles in the regulation of systemic glucose metabolism by activating the ERBB3-mediated signalling pathways, although little information is available on which *Nrg1* isoforms are expressed in the liver and their functions as a local mediator of hepatic glucose metabolism.

In the present study, we have demonstrated that the type I isoform of NRG1 with an α-type EGF-like domain (NRG1α) suppresses hepatic glucose production, in part, by activating both the AKT and ERK signalling cascades and subsequently inhibiting FoxO1-mediated gluconeogenic gene expression. These results suggest that NRG1α acts as a local regulator of liver gluconeogenesis in an autocrine/paracrine manner.

## Results

### *Type I Nrg1α* is a major isoform of *Nrg1* in normal mouse liver

**Figure 1:**
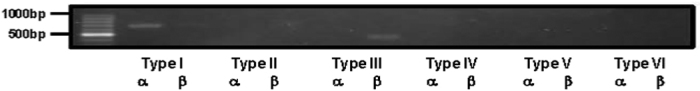
*Type I Nrg1α* is predominantly expressed in normal liver. The expression pattern of *Nrg1* isoforms was analysed by PCR. Note that two distinct types of *Nrg1* transcripts, *Type I Nrg1α* and *Type III Nrg1β*, were detected in normal liver.

NRG1 has been reported to produce six N-terminal types of proteins with various isoforms using distinct promoters and alternative splicing^15^. To identify which isoforms of *Nrg1* are expressed in mouse liver, we performed a conventional PCR using specific primer sets and determined the sequences of the PCR products. Two distinct types of *Nrg1, Type I Nrg1α* and *Type III Nrg1β*, were predominantly detected in the liver by PCR (Fig. 1 and Supplementary Fig. S1). However, repeated sequencing of the latter isoform revealed that exon sequences encoding its N-terminal domain were not identical to those reported in the brain^16^ and lacked the final 70 nucleotides of exon 1, introducing premature stop codons in the EGF-like domain of Type III *Nrg1*β. As the EGF-like domain is required for biological functions via binding to its receptors, Type I NRG1α (designated NRG1α) is likely the only physiologically active NRG1 isoform in mouse liver.

NRG1 is proteolytically cleaved to locally release its extracellular region containing the EGF-like domain^6^. We next determined whether the N-terminal ectodomain of hepatic NRG1α is released locally using isolated primary hepatocytes. Endogenous NRG1α protein was barely detectable in whole cell extracts but was found to be constitutively cleaved and released its N-terminal ectodomain (~45 kDa) in the concentrated conditioned medium of hepatocytes (Supplementary Fig. S2a). Consistent with this result, cleaved NRG1α fragment was enriched in the conditioned medium with small amounts of both full-length (~100 kDa) and cleaved (~45 kDa) form of NRG1α in the cell lysate when hepatocytes were infected with a *Nrg1α*-expressing adenovirus (Supplementary Fig. S2b). These results support the hypothesis that NRG1α acts as a local mediator in an autocrine and/or paracrine manner in liver.

### Forced *Nrg1α* expression in liver improves systemic glucose disposal and decreases hepatic gluconeogenesis

To examine effects of hepatic NRG1α overexpression in regulating glucose metabolism, we injected plasmids encoding mouse *Nrg1α* tagged with a C-terminal Flag peptide into mouse livers three times every 6 days by hydrodynamic tail vein injection. Two days after the last injection, NRG1α protein was detected at ~100 kDa exclusively in the liver but not other tissues, such as skeletal muscle and epididymal adipose tissue using an anti-Flag antibody (Supplementary Fig. S3a). Consistent with the results of cells overexpressing *Nrg1α*, the cleaved form of NRG1α was predominantly observed in liver treated with the *Nrg1α*-expressing plasmid but not an empty vector using an anti-NRG1α antibody (Supplementary Fig. S3b). These results suggest that normal mouse liver shows limited expression of NRG1α at basal conditions, and that we have succeeded to overexpress NRG1α predominantly in the liver in the same manner to endogenously expressed NRG1α.

**Figure 2:**
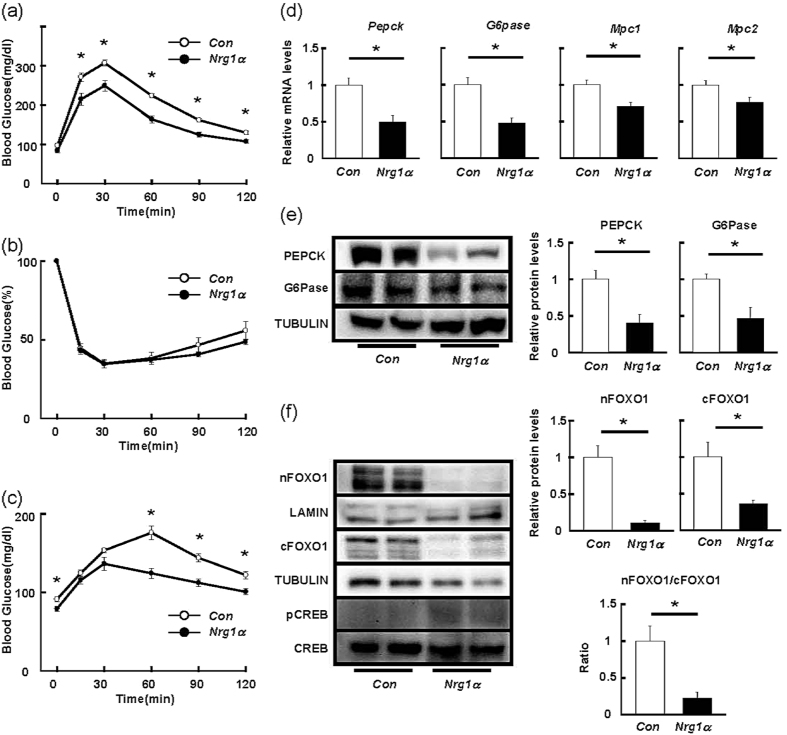
Forced *Nrg1α* expression in the liver improves systemic glucose disposal and suppresses hepatic glucose production. OGTT (**a**, n = 8), ITT (**b**, n = 6), and PTT (**c**, n = 8) in mice overexpressing *Nrg1α* in liver. Analyses of the expression of hepatic gluconeogenesis-related genes in *Nrg1α-*overexpressing mice (**d**, n = 10). Representative immunoblots of PEPCK and G6Pase in whole liver (**e**), and those of nuclear (nFOXO1) and cytosolic (cFOXO1) FOXO1 and nuclear phospho-CREB (pCREB) in the liver (**f**). The results of densitometric quantification are shown at the right. n = 6 mice per group in (**e**,**f**). *P < 0.05. Con, empty pCAGGS vector; Nrg1α, mouse *Nrg1α*-expressing vector.

Systemic glucose disposal after oral glucose challenge (OGTT) was significantly enhanced in mice overexpressing *Nrg1α* compared to that of mice receiving the empty vector (Fig. 2a), whereas this treatment had little, if any, effect on insulin sensitivity in the peripheral tissues (Fig. 2b). To confirm the effects of hepatic NRG1α overexpression on peripheral insulin signalling, we measured the phosphorylation levels of AKT in response to insulin in skeletal muscle and epididymal adipose tissue. Overexpression of hepatic *Nrg1α* did not show any impacts on insulin-induced phosphorylation of AKT in these tissues (Supplementary Fig. S4). Forced expression of *Nrg1α* in the liver also attenuated hepatic gluconeogenic activity with decreased fasting glucose levels in pyruvate tolerance test (PTT) (Fig. 2c). NRG1 exerts its biological functions by binding to two single-transmembrane receptor tyrosine kinases, ERBB3 and ERBB4^5^. The *Nrg1α*-induced inhibitory action on the hepatic glucose production was tightly associated with selective phosphorylation of ERBB3 in the liver overexpressing *Nrg1α* (Supplementary Fig. S5). In contrast, we hardly detected ERBB4 phosphorylation in these mice. These results suggested that NRG1α improves systemic glucose tolerance mainly by suppressing hepatic glucose production but not by increasing insulin sensitivity in the peripheral organs in an ERBB3-dependent manner. Consistent with this hypothesis, mRNA levels of the key gluconeogenic enzymes, *Pepck* and *G6pase*, and mitochondrial transporters for pyruvate, *Mpc1* and *Mpc2*, were reduced in *Nrg1α*-overexpressing livers (Fig. 2d). We also confirmed the *Nrg1α*-mediated decrease in protein levels of PEPCK and G6Pase in the mice (Fig. 2e). FoxO1 and CREB are crucial transcription factors for hepatic glucose production under fasting conditions. *Nrg1α* reduced both nuclear and cytoplasmic amounts of FoxO1 with a marked decrease in ratio of nuclear to cytoplasmic FoxO1 (Fig. 2f). On the other hand, *Nrg1α* showed marginal effect on CREB phosphorylation. Taken together, these results suggest that NRG1α ameliorates systemic glucose tolerance by suppressing FoxO1-mediated gluconeogenesis in the liver.

### *Nrg1α* reduces glucose production with suppressed gluconeogenic gene induction in primary hepatocytes

**Figure 3:**
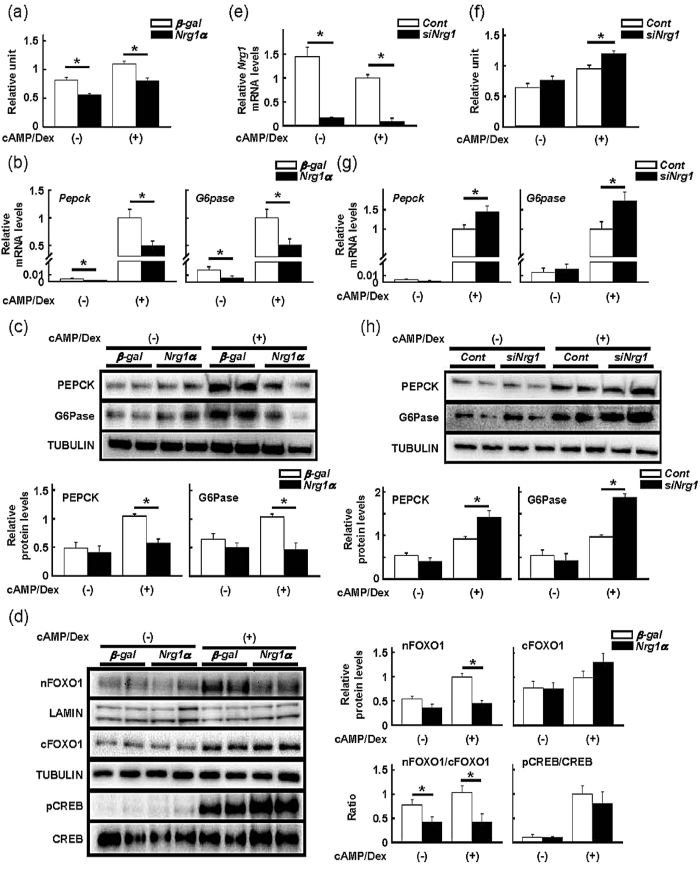
Hepatic glucose production is attenuated in isolated primary hepatocytes expressing *Nrg1α*. (**a**) Glucose production of hepatocytes infected with the *Nrg1α*-expressing adenovirus. n = 4 per group. *P < 0.05. (**b**) Quantification of *Pepck* and *G6pase* mRNA in *Nrg1α*-expressing hepatocytes stimulated with or without 8-bromo-cAMP and dexamethasone. n = 4 (without cAMP/Dex) and 6 (with cAMP/Dex) per group. *P < 0.05. (**c**) Representative immunoblots of PEPCK and G6Pase in *Nrg1α*-expressing hepatocytes. The results of densitometric quantification are shown. n = 4 mice per group. *P < 0.05. (**d**) Representative immunoblots of FOXO1 and phospho-CREB in the nuclear fraction of *Nrg1α*-expressing hepatocytes. nFOXO1: nuclear FOXO1, cFOXO1: cytosolic FOXO1. The results of densitometric quantification are shown at the right. n = 4 mice per group. *P < 0.05. (**e**) Validation of siRNA for *Nrg1* gene in hepatocytes. n = 6 per group. *P < 0.05. (**f**) Glucose production of hepatocytes silenced endogenous *Nrg1α* expression. n = 6 per group. *P < 0.05. (**g**) Quantification of *Pepck* and *G6pase* mRNA in *Nrg1α*-silencing hepatocytes stimulated with or without 8-bromo-cAMP and dexamethasone. n = 6 per group. *P < 0.05. (**h**) Representative immunoblots of PEPCK and G6Pase in *Nrg1α*-silencing hepatocytes. The results of densitometric quantification are shown. n = 4 mice per group. *P < 0.05.

To further confirm the inhibitory effects of NRG1α on glucose production, we incubated isolated primary hepatocytes in medium containing gluconeogenic substrates and simultaneously stimulated the cells with cAMP and dexamethasone. This treatment modestly but significantly increased glucose output from the hepatocytes, but adenoviral-mediated *Nrg1α* overexpression reduced both basal and stimulated hepatic glucose production (Fig. 3a). Consistent with these results, the induction of PEPCK and G6Pase at their transcript and protein levels by the treatment with cAMP and dexamethasone was suppressed to a greater extent in hepatocytes overexpressing *Nrg1α* (Fig. 3b,c). Additionally, nuclear FoxO1 accumulation was reduced without any impacts on cytosolic FoxO1 in these cells (Fig. 3d).

We next examined if suppression of endogenous *Nrg1* expression could conversely enhance glucose production in hepatocytes. Silencing *Nrg1* expression by siRNA modestly but significantly increased glucose production with higher levels of both transcript and protein levels of PEPCK and G6Pase only when hepatocytes were stimulated with cAMP and dexamethasone (Fig. 3e,f,g,h), further strengthening the importance of endogenously expressed NRG1α on hepatic gluconeogenesis.

### ERK and AKT mediate NRG1α-induced inhibition of hepatic gluconeogenesis via ERBB3 phosphorylation

**Figure 4:**
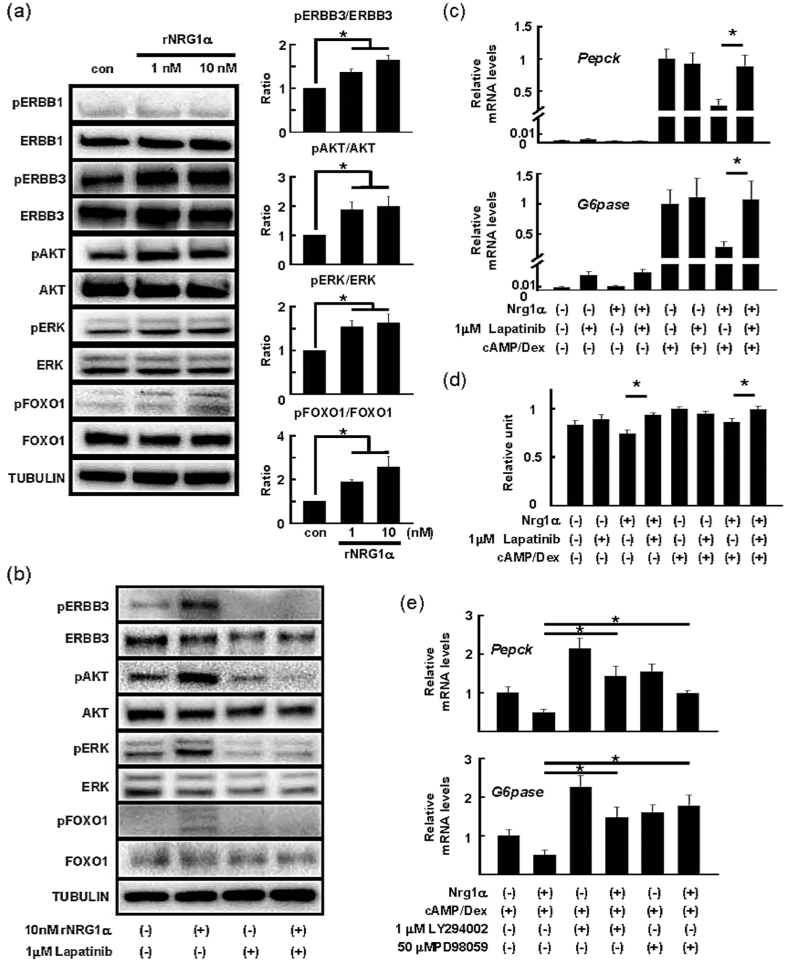
The recombinant EGF-like domain of NRG1α activates ERBB3 and its downstream signalling cascades, ERK and AKT. (**a**) Representative immunoblots and densitometric quantification of ERBB signalling cascades in hepatocytes stimulated with rNRG1α. Normalized levels in hepatocytes without rNRG1α treatment in each experiment were expressed as 1. n = 6 per each group. *P < 0.05. (**b**) Representative immunoblots of ERBB signalling cascades in rNRG1α-stimulated hepatocytes treated with lapatinib. (**c**) Quantification of *Pepck* and *G6pase* mRNA in *Nrg1α*-expressing hepatocytes treated with lapatinib. n = 6 per group. *P < 0.05. (**d**) Glucose production of *Nrg1α*-expressing hepatocytes treated with lapatinib. n = 6 per group. *P < 0.05. (**e**) Quantification of *Pepck* and *G6pase* mRNA in *Nrg1α*-overexpressing hepatocytes treated with ERBB signalling cascade inhibitors. n = 6 (without inhibitor) and 4 (with inhibitor) per group. *P < 0.05.

NRG1 undergoes proteolytic cleavage at the juxtamembrane region and subsequently releases its diffusible ectodomain, which has a biologically active EGF-like domain^5^. The ectodomain of NRG1 binds to either ERBB3 or ERBB4 to express its functions on target cells and tissues^5^. To assess the signalling cascade of NRG1α in the liver, we treated hepatocytes with bacterially expressed NRG1α recombinant protein (rNRG1α) that contains an α-type EGF-like domain. Transient treatment (30 min) of isolated hepatocytes with rNRG1α resulted in increased phosphorylation of ERBB3, but not ERBB1, in a dose-dependent manner (Fig. 4a). On the other hand, expression of both ERBB2 and ERBB4 was barely detectable in our experiments (data not shown). rNRG1α also activated two ERBB receptor-associated downstream kinases, AKT and ERK, and increased FoxO1 phosphorylation (Fig. 4a).

To further confirm the importance of these NRG1α-activated signalling cascades in suppressing hepatic gluconeogenic activity, we assessed whether pharmacological inhibition of the ERBB3-dependent PI3K-AKT and Raf-MEK-ERK pathways could reverse the rNRG1α-elicited suppression of *Pepck* and *G6pase* induction. As ERBB3 shows little, if any, kinase activity, ERBB3 requires dimerization with another ERBB receptor such as ERBB1 and ERBB2 to transmit its signals into cells^17^. We used lapatinib, a specific inhibitor for both ERBB1 and ERBB2, to evaluate the significance of ERBB3-mediated pathway in the NRG1α-evoked inhibition of hepatic gluconeogenesis. As expected, the pretreatment with lapatinib completely abolished both basal and rNRG1α-dependent ERBB3 phosphorylation (Fig. 4b). This is accompanied by a marked decrease in the phosphorylated forms of AKT, ERK, and FoxO1 (Fig. 4b). In addition, such a treatment totally restored the *Nrg1α*-induced suppression of expression of *Pepck* and *G6pase* and hepatic glucose production in hepatocytes stimulated with cAMP and dexamethasone (Fig. 4c,d). In addition, LY294002 (1 μM) and PD98059 (50 μM) completely inhibited both basal and the rNRG1α-elicited AKT and ERK phosphorylation, respectively (Supplementary Fig. S6). Both inhibitors at the same concentrations substantially but not completely abolished the inhibitory effects of *Nrg1α* on *Pepck* and *G6pase* induction (Fig. 4e).

### Acute rNRG1α treatment suppresses elevation of blood glucose levels in OGTT and PTT

**Figure 5:**
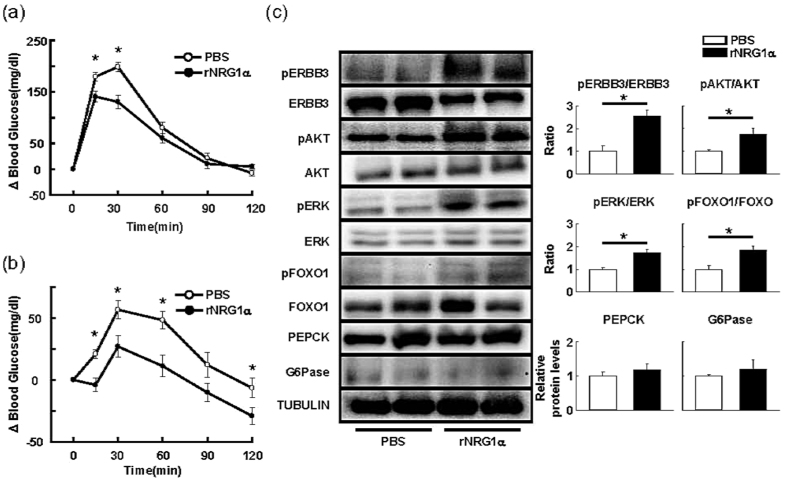
The recombinant EGF-like domain of NRG1α reduces elevation of blood glucose levels in OGTT and PTT. OGTT (**a**, n = 7) and PTT (**b**, n = 8) were performed using mice received a single injection of rNRG1α (i.p.; 100 ng/g body weight). *P < 0.05 compared with mice received PBS. (**c**) Representative immunoblots of ERBB signalling cascades in liver treated with rNRG1α. The results of densitometric quantification are shown. n = 4 mice per group. *P < 0.05.

We next examined whether a short-term exposure (15 min before glucose and pyruvate challenge) to rNRG1α also shows its blood glucose-lowering effects. A single injection of rNRG1α successfully increased systemic glucose disposal and attenuated hepatic glucose production (Fig. 5a,b), suggesting that NRG1α has direct inhibitory effects on the regulation of glycolytic/gluconeogenic fluxes in the liver. These alterations were accompanied by activation of ERBB3-AKT/ERK-FoxO1 signalling cascade, but not any changes in PEPCK and G6Pase expression (Fig. 5c).

### A liver-specific deletion of *Nrg1* gene shows little alterations on blood glucose levels after glucose and pyruvate challenge

To determine effects of a loss of hepatic *Nrg1* gene on systemic glucose metabolism, we generated liver-specific *Nrg1* gene knockout mice (Nrg1KO) and subjected to OGTT and PTT. Unexpectedly and inconsistent with our *in vitro* results, Nrg1KO mice were found to display no apparent difference in fasting blood glucose levels compared to wild-type (WT) mice and elevate blood glucose levels after glucose and pyruvate challenge comparable to WT mice (Supplementary Fig. S7), suggesting little, if any, effect of hepatic *Nrg1* on systemic glucose metabolism under non-stressed conditions.

## Discussion

Gluconeogenesis is a critical metabolic pathway in hepatic glucose homeostasis, especially in starvation conditions. Aberrant activation of gluconeogenesis occurs due to hepatic insulin resistance and is a hallmark of diabetes mellitus^18^. Various hormones, including insulin and glucagon, respond to alterations in nutritional conditions and contribute to the coordinated regulation of hepatic glucose production. As the liver is a major secretory organ, hepatocytes control their own glucose metabolism by releasing local mediators. However, information is limited on the biological significance of liver-derived humoral factors in hepatic glucose metabolism. In the present study, we demonstrated that Type I NRG1α is exclusively expressed among various NRG1 isoforms in mouse liver and may function as a local mediator to control hepatic gluconeogenesis by releasing its N-terminal ectodomain extracellularly. The NRG1α-dependent inhibition of hepatic glucose production occurs by binding to ERBB3 and subsequently, in part, activating the ERK and AKT signalling pathways, resulting in decreased expression of key gluconeogenic genes.

NRG1 produces multiple isoforms and exerts its diverse biological functions in an autocrine and/or paracrine manner in various organs^6^. Among these isoforms, mouse liver predominantly expresses Type I NRG1α which is constitutively cleaved to release its N-terminal portion containing an α-type EGF-like domain extracellularly as a functional ligand for ERBB receptors. Although NRG1 can bind with and activate both ERBB3 and ERBB4 receptor on target cells, Type I NRG1α is likely to show a higher affinity to ERBB3 than ERBB4 in liver given that ERBB3 is exclusively phosphorylated in both liver overexpressing *Type I NRG1α* and primary hepatocytes exposed to rNRG1α. This might be explained by the differences in hepatic expression levels of ERBBs or Type I NRG1α-specific affinity for ERBB3. ERBB3 and ERBB1 are highly expressed in rat liver, whereas ERBB2 and ERBB4 expression are hardly detectable^12^. Consistent with this report, we also found that ERBB3 protein is abundantly present in mouse liver, supporting the hypothesis that NRG1α preferentially recognizes and activates ERBB3 receptor in the liver. On the other hand, the latter hypothesis is also conceivable when considering that different type of NRGs exhibits distinct pattern of ERBB receptor activation^19^. For example, Neuregulin4, another member of the neuregulin family, has been reported to regulate hepatic lipogenesis by binding to hepatic ERBB4, but not ERBB3 receptor^20^. In addition, variation in the short C-terminal region of the EGF-like domain, α and β type, in NRG1 also can affect binding capacity for and activation of ERBB receptors. NRG1β displays a higher binding ability for almost all ERBB receptors complexes containing either ERBB3 or ERBB4, whereas α type EGF-like domain of NRG1 can transmit its signal efficiently via binding exclusively with ERBB2/3 receptor complex^21^. This is further supported by other reports showing that ERBB3 kinase activity is too weak to phosphorylate via formation of ERBB3 homodimers without any interaction with other ERBB receptors and small amounts of ERBB2 are sufficient to phosphorylate and activate ERBB3 receptor via a transient formation of ERBB2/3 receptor complex^22^. Although we did not detect any expression of ERBB2 protein in isolated mouse hepatocytes and liver, trace amounts of ERBB2 seem to be involved in NRG1α-mediated ERBB3 activation and subsequent responses in the regulation of gluconeogenesis in the liver. In fact, we found that the treatment with lapatinib successfully inhibits rNRG1α-elicited ERBB3 phosphorylation with a marked suppression of phosphorylation of its downstream molecules such as AKT, ERK, and FoxO1. In addition, such a treatment also restores expression of gluconeogenic genes and hepatic glucose production in hepatocytes overexpressing *Nrg1α*. When considering that ERBB1 is barely phosphorylated in response to rNRG1α despite its ample expression, our present results suggest a crucial role of ERBB2/3 as functional receptor complex for Type I NRG1α in liver. Furthermore, an immunoglobulin-like domain of NRG1α, another structural characteristic of Type I NRG1s present in the N-terminal of the EGF-like domain, might compensate for the lower activity of NRG1α by increasing its local concentrations through heparan sulphate binding on target cells, enhancing its ability to stimulate ERBB receptors^23^. Therefore, our results strongly suggest the importance of hepatic Type I NRG1α as a local mediator in the regulation of glucose metabolisms by activating ERBB3 receptor in mouse liver, as described below.

Our current results clearly demonstrated that hepatic NRG1α attenuates elevation of blood glucose levels after both glucose and pyruvate challenge by supressing hepatic glucose production. This is consistent with previous reports showing that acute injection of NRG1β improved glucose tolerance in rodents^12^,^14^. López-Soldado *et al*. argued that a NRG1β1-dependent increase in hepatic glucose utilization with enhanced glycolysis and glycogen synthesis may contribute to elevated systemic glucose clearance by activating the hepatic AKT signalling cascade^12^. On the other hand, Ennequin *et al*. pointed out the AKT-FoxO1 pathway-mediated inhibitory effects of NRG1β2 on hepatic gluconeogenesis, presumably leading to stimulated lactate production and consequently decreased hepatic glucose production^14^. These could explain the acute effects of rNRG1α on systemic glucose metabolism, as a single injection of rNRG1α into mice also shows its blood glucose-lowering effects after glucose and pyruvate challenge by stimulating the ERBB3-AKT signalling cascade without any changes in PEPCK and G6Pase protein levels. In our experiments, we also revealed that chronic *Nrg1α* overexpression in the liver improves systemic glucose disposal with reduced hepatic glucose production by suppressing expressions of key gluconeogenic genes such as *Pepck* and *G6pase*. This is in line with a previous report showing enhanced systemic glucose clearance in diabetic mice by long-term exposure to NRG1β2, although any direct evidence for the NRG1-mediated suppression of liver gluconeogenesis is not provided^14^. Chronic *Nrg1α* overexpression in the liver decreases hepatic FoxO1 transcript levels (data not shown), suggesting a possible involvement of NRG1α-mediated transcriptional regulation of FoxO1 in the reduced hepatic gluconeogenesis. However, considering that an intensive reduction of nuclear FoxO1 compared to cytosolic one occurs by *Nrg1α*, sustained activation of ERBB3-AKT-FoxO1 signalling cascade seems to play crucial roles in the NRG1α-evoked inhibition of hepatic glucose production in mouse liver. Consistent with this hypothesis, we found that *Nrg1α* attenuates not only expression of PEPCK and G6Pase but also glucose production in isolated hepatocytes with enhanced cytoplasmic sequestration of FoxO1 but not any changes in FoxO1 transcript levels (data not shown). In addition, silencing of hepatic *Nrg1α* conversely enhances glucose production with increased these gluconeogenic enzymes expression in cAMP/dexamethasone-stimulated hepatocytes, further supporting the importance of NRG1α in the regulation of hepatic gluconeogenesis. Moreover, the ERBB3 signalling pathways stimulates not only the PI3K-AKT cascade but also Raf-MEK-ERK in the liver. Therefore, the modest, but significant, phosphorylation of ERK by NRG1α may cause phosphorylation and subsequent inactivation of FoxO1^24^. Indeed, this hypothesis is further supported by our current findings that an ERK inhibitor partially but substantially abolishes *Nrg1α*-induced suppression of gluconeogenic enzyme expression. Taken together, these results strongly suggest that NRG1α inhibits hepatic glucose production, at least in part, in both an AKT- and ERK-dependent manner by suppressing FoxO1 activity.

Although a liver-specific deletion of *Nrg1* gene did not evoke any alterations in systemic glucose disposal after glucose and pyruvate challenge, our data provide an evidence that Type I NRG1α might act as a local mediator to control gluconeogenesis by activating ERBB3 signalling cascade and consequently inactivating FoxO1-mediated gene expression in the liver. Hepatic NRG1 expression has been reported to be induced in diabetic rats, suggesting the pathological importance of NRG1α in the regulation of liver glucose metabolism in insulin resistant conditions^12^. Although further investigation is needed to examine its pathological significance, our findings indicate that NRG1α may have therapeutic potential in reducing hepatic glucose production in diabetes mellitus.

## Methods

### Animals and treatments

C57BL6/J male mice (5–6 weeks old) were obtained from CLEA Japan (Tokyo, Japan). Liver-specific Nrg1 knockout mice (male, 5–6 weeks old) were generated by crossing Nrg1 mutant mice (Acc. No. CDB0743K; http://www.clst.riken.jp/arg/mutant%20mice%20list.html)^25^ and Alb-Cre transgenic mice^26^. These mice were maintained on a 12/12 h light/dark cycle with free access to water and chow (Sankyo Labo Service, Tokyo, Japan). All experiments were conducted in accordance with the Waseda University Animal Welfare Guidelines and were approved by the Animal Experimentation Committee of the Waseda University (Protocol number: 2015-A083a). Mice received 30 μg of either pCAGGS alone or a mouse *Nrg1α*-expressing vector intravenously by hydrodynamic tail vein injection^27^. The mice were injected three times every 6 days. Two days after the last injection, mice were subjected to further analyses. OGTT (2 mg/g body weight), PTT (2 mg/g body weight, intraperitoneal injection), and insulin tolerance test (ITT; 0.75 IU/Kg body weight, intraperitoneal injection) were performed on mice fasted for 16 h, 24 h, and 6 h, respectively. Blood glucose levels were measured using an automatic glucose monitor (Accu-Chek, Roche Diagnostics, Basel, Switzerland). In some experiments, recombinant mouse NRG1α (rNRG1α) protein (100 ng/g body weight) was intraperitoneally administered to mice 15 min before performing the OGTT and PTT.

### PCR and quantitative PCR (qPCR)

Complementary DNA was synthesized using total RNA extracted from the liver and isolated hepatocytes with a GoScript^TM^ Reverse transcription system (Promega, Fitchburg, WI, USA), as described previously^28^. A conventional PCR was performed using specific primer sets (Supplemental Table 1) and Q5 High-Fidelity DNA Polymerase (New England BioLabs, Ipswich, MA, USA) to determine which isoforms of *Nrg1* were expressed in mouse liver. mRNA levels were measured using either GoTaq qPCR Master Mix (Promega) or FastStart TaqMan^®^ Probe Master (Thermo Fisher Scientific, Waltham, MA, USA). TaqMan gene expression assays (Thermo Fisher Scientific) and sequences of primers and probe are listed in Supplemental Table 1. Gene expression levels were normalized to *18S rRNA* as an internal control.

### Recombinant NRG1α protein fragment preparation

A partially purified rNRG1α fragment from His^171^ to Arg^242^ of the mouse NRG1α protein was prepared using pCold I (TaKaRa Bio, Shiga, Japan) and HisTALON gravity columns (TaKaRa Bio).

### Western blotting

Western blotting analysis was performed using 20–100 μg of either nuclear extracts, cytoplasmic extracts, or whole extracts of mouse livers and isolated hepatocytes, as described previously^26^. The conditioned medium of isolated hepatocytes was also analysed. Antibodies against the following proteins were used: AKT, phospho-AKT (Ser^473^), CREB, phospho-CREB (Ser^133^), ERBB1, phospho-ERBB1 (Tyr^1068^), ERBB3, phospho-ERBB3 (Tyr^1289^), ERK1/2, phospho-ERK1/2 (Thr^202^/Tyr^204^), FoxO1, phospho-FoxO1 (Ser^256^) (Cell Signaling Technology, Danvers, MA, USA), NRG1α (R&D Systems, Minneapolis, MN, USA), Lamin A/C (Santa Cruz Biotechnology, Santa Cruz, CA, USA), α-Tubulin and Flag (Sigma, St. Louis, MO, USA). Polyclonal antibody against mouse G6Pase and PEPCK was originally established by injecting the corresponding peptides (G6Pase; aa344-357, PEPCK; aa587-600) into guinea pigs (TaKaRa Bio).

### Cell culture, adenovirus infection, and rNRG1α treatment

Primary mouse hepatocytes were cultured in William’s medium E (Thermo Fisher Scientific) supplemented with 10% foetal bovine serum at 37 °C in 5% CO_2_. Cells were treated with either an adenovirus (ViraPower Adenoviral Gateway Expression Kit, Thermo Fisher Scientific) expressing mouse *Nrg1α* or siRNA (Supplemental Table 1, Integrated DNA Technologies KK, Tokyo, Japan) targeting for mouse *Nrg1* 24 h before stimulation with 500 μM 8-bromo-cAMP (Cayman Chemical, Ann Arbor, MI, USA) and 100 nM dexamethasone (Sigma) for 3 h. Viruses encoding *β-galactosidase* were used as a control. A multiplicity of infection of 50 for each virus was used for the infection. In some experiments, cells were pretreated with rNRG1α (1 or 10 nM) 30 min, or inhibitors such as Ly294002 (Wako Pure Chemical Industries), PD98059 (Cayman Chemical) or lapatinib (BioVision, Inc, Milpitas, CA, USA) 1 h before the stimulation. The conditioned media (24 h incubation) of isolated hepatocytes were collected to determine whether the N-terminal region of NRG1 endogenously expressed in hepatocytes was cleaved and released.

### Glucose production assay

Primary mouse hepatocytes were incubated for 6 h in KRB buffer (119 mM NaCl, 5 mM KCl, 2.6 mM KH_2_PO_4_, 2.6 mM MgSO_4_, 2 mM CaCl_2_, 24.6 mM NaHCO_3_ and 10 mM HEPES, pH 7.4) supplemented with 0.5% BSA and gluconeogenic substrates (10 mM sodium lactate and 5 mM sodium pyruvate) in the presence or absence of 500 μM 8-bromo-cAMP and 100 nM dexamethasone. The conditioned medium was collected to measure glucose concentrations using Accu-Chek. Glucose production was normalized to the total hepatocyte protein concentrations.

### Statistical analyses

Results are expressed as the mean ± SEM. Statistical analyses were performed using Mann-Whitney U tests and one-way ANOVAs, followed by Tukey-Kramer tests for all experiments. *P* values < 0.05 were considered to be significant.

## Additional Information

**How to cite this article**: Arai, T. *et al*. Type I neuregulin1α is a novel local mediator to suppress hepatic gluconeogenesis in mice. *Sci. Rep.* **7**, 42959; doi: 10.1038/srep42959 (2017).

**Publisher's note:** Springer Nature remains neutral with regard to jurisdictional claims in published maps and institutional affiliations.

## Supplementary Material

Supplementary Information

## References

[b1] Liu T. Y. . Irisin inhibits hepatic gluconeogenesis and increases glycogen synthesis via the PI3K/Akt pathway in type 2 diabetic mice and hepatocytes. Clin Sci (Lond) 129, 839–850, doi: 10.1042/CS20150009 (2015). 26201094

[b2] Combs T. P. & Marliss E. B. Adiponectin signaling in the liver. Rev Endocr Metab Disord 15, 137–147, doi: 10.1007/s11154-013-9280-6 (2014). 24297186 PMC4152934

[b3] Pal D. . Fetuin-A acts as an endogenous ligand of TLR4 to promote lipid-induced insulin resistance. Nature Medicine 18, 1279–1285, doi: 10.1038/nm.2851 (2012). 22842477

[b4] Misu H. . A Liver-Derived Secretory Protein, Selenoprotein P, Causes Insulin Resistance. Cell Metabolism 12, 483–495, doi: 10.1016/j.cmet.2010.09.015 (2010). 21035759

[b5] Guma A., Martinez-Redondo V., Lopez-Soldado I., Canto C. & Zorzano A. Emerging role of neuregulin as a modulator of muscle metabolism. American Journal of Physiology-Endocrinology and Metabolism 298, E742–E750, doi: 10.1152/ajpendo.00541.2009 (2010). 20028964

[b6] Falls D. L. Neuregulins: functions, forms, and signaling strategies. Experimental Cell Research 284, 14–30, doi: 10.1016/S0014-4827(02)00102-7 (2003). 12648463

[b7] Meyer D. & Birchmeier C. Multiple essential functions of neuregulin in development. Nature 378, 386–390, doi: 10.1038/378386a0 (1995). 7477375

[b8] Sudol M. Neuregulin 1-activated ERBB4 as a “dedicated” receptor for the Hippo-YAP pathway. Science Signaling 7, pe29, doi: 10.1126/scisignal.aaa2710 (2014). 25492964

[b9] Pan B., Huang X. F. & Deng C. Antipsychotic treatment and neuregulin 1-ErbB4 signalling in schizophrenia. Prog Neuropsychopharmacol Biol Psychiatry 35, 924–930, doi: 10.1016/j.pnpbp.2011.04.002 (2011). 21513767

[b10] Canto C. . Neuregulins mediate calcium-induced glucose transport during muscle contraction. J Biol Chem 281, 21690–21697, doi: 10.1074/jbc.M600475200 (2006). 16740635

[b11] Canto C. . Neuregulin signaling on glucose transport in muscle cells. Journal of Biological Chemistry 279, 12260–12268, doi: 10.1074/jbc.M308554200 (2004). 14711829

[b12] Lopez-Soldado I. . Neuregulin improves response to glucose tolerance test in control and diabetic rats. Am J Physiol Endocrinol Metab 310, E440–451, doi: 10.1152/ajpendo.00226.2015 (2016). 26714846

[b13] Caillaud K. . Neuregulin 1 improves glucose tolerance in adult and old rats. Diabetes Metab 42, 96–104, doi: 10.1016/j.diabet.2015.08.003 (2016). 26404652

[b14] Ennequin G. . Neuregulin 1 Improves Glucose Tolerance in db/db Mice. PLoS One 10, e0130568, doi: 10.1371/journal.pone.0130568 (2015). 26230680 PMC4521942

[b15] Liu X. . Specific regulation of NRG1 isoform expression by neuronal activity. J Neurosci 31, 8491–8501, doi: 10.1523/JNEUROSCI.5317-10.2011 (2011). 21653853 PMC3154699

[b16] Ho W. H., Armanini M. P., Nuijens A., Phillips H. S. & Osheroff P. L. Sensory and motor neuron-derived factor. A novel heregulin variant highly expressed in sensory and motor neurons. J Biol Chem 270, 14523–14532, doi: 10.1074/jbc.270.24.14523 (1995). 7782315

[b17] Sliwkowski M. X. . Coexpression of erbB2 and erbB3 proteins reconstitutes a high affinity receptor for heregulin. J Biol Chem 269, 14661–14665 (1994). 7514177

[b18] Brown M. S. & Goldstein J. L. Selective versus total insulin resistance: A pathogenic paradox. Cell Metabolism 7, 95–96, doi: 10.1016/j.cmet.2007.12.009 (2008). 18249166

[b19] Hobbs S. S. . Neuregulin isoforms exhibit distinct patterns of ErbB family receptor activation. Oncogene 21, 8442–8452, doi: 10.1038/sj.onc.1205960 (2002). 12466964

[b20] Wang G. X. . The brown fat-enriched secreted factor Nrg4 preserves metabolic homeostasis through attenuation of hepatic lipogenesis. Nature Medicine 20, 1436–1443, doi: 10.1038/nm.3713 (2014). PMC4257907 25401691

[b21] Pinkas-Kramarski R. . ErbB tyrosine kinases and the two neuregulin families constitute a ligand-receptor network. Mol Cell Biol 18, 6090–6101, doi: 10.1128/MCB.18.10.6090 (1998). 9742126 PMC109195

[b22] Steinkamp M. P. . erbB3 is an active tyrosine kinase capable of homo- and heterointeractions. Mol Cell Biol 34, 965–977, doi: 10.1128/MCB.01605-13 (2014). 24379439 PMC3958038

[b23] Eto K., Eda K., Kanemoto S. & Abe S. The immunoglobulin-like domain is involved in interaction of Neuregulin1 with ErbB. Biochem Biophys Res Commun 350, 263–271, doi: 10.1016/j.bbrc.2006.09.028 (2006). 17007820

[b24] Jiao P., Feng B. & Xu H. Mapping MKP-3/FOXO1 interaction and evaluating the effect on gluconeogenesis. PLoS One 7, e41168, doi: 10.1371/journal.pone.0041168 (2012). 22848439 PMC3405053

[b25] Zhang J. . Neuregulins are essential for spermatogonial proliferation and meiotic initiation in neonatal mouse testis. Development 138, 3159–3168, doi: 10.1242/dev.062380 (2011). 21715427

[b26] Nishiyama Y. . HIF-1 alpha induction suppresses excessive lipid accumulation in alcoholic fatty liver in mice. Journal of Hepatology 56, 441–447, doi: 10.1016/j.jhep.2011.07.024 (2012). 21896344

[b27] Gao M., Ma Y., Cui R. & Liu D. Hydrodynamic delivery of FGF21 gene alleviates obesity and fatty liver in mice fed a high-fat diet. J Control Release 185, 1–11, doi: 10.1016/j.jconrel.2014.03.047 (2014). 24747761 PMC4159144

[b28] Ochiai D. . Disruption of HIF-1alpha in hepatocytes impairs glucose metabolism in diet-induced obesity mice. Biochem Biophys Res Commun 415, 445–449, doi: 10.1016/j.bbrc.2011.10.089 (2011). 22051049 PMC6592821

