# Supplementary material for: Type I neuregulin1α is a novel local mediator to suppress hepatic gluconeogenesis in mice
Source: Sci Rep. 2017 Feb 20;7:42959. doi: 10.1038/srep42959 (PMC5317163; doi:10.1038/srep42959)
Supplement: Supplementary Information [file srep42959-s1.pdf]

Supplementary Information

Type I neuregulin1α is a novel local mediator to suppress hepatic gluconeogenesis in mice

Takatomo Arai[1], Yumika Ono[1], Yujiro Arimura[1], Keimon Sayama[1], Tomohiro Suzuki[1], Satoko Shinjo[1], Mai Kanai[1], Shin-ichi Abe[2], Kentaro Semba[1], Nobuhito Goda [1*]

[1]Department of Life Science and Medical Bioscience, School of Advanced Science and Engineering, Waseda University, Tokyo, 162-8480, Japan; [2]Center for General Education, Kumamoto Health Science University, Kumamoto, 861-5598, Japan

*Correspondence and requests for materials should be addressed to N.G. (e-mail: goda@waseda.jp)

```
Beta            MSERKEGRGKGKGKKKDRGSRGKPAPAEGDPSPALPPRLKEMKSQESAAGSKLVLRCETS
Alpha           MSERKEGRGKGKGKKKDRGSRGKPAPAEGDPSPALPPRLKEMKSQESAAGSKLVLRCETS
Human           MSERKEGRGKGKGKKKERGSGKKPESAAGSQSPALPPRLKEMKSQESAAGSKLVLRCETS
                ****************:***  ** .* *. *****************************

Beta            SEYSSLRFKWFKNGNELNRRNKPQNVKIQKKPGKSELRINKASLADSGEYMCKVISKLGN
Alpha           SEYSSLRFKWFKNGNELNRRNKPQNVKIQKKPGKSELRINKASLADSGEYMCKVISKLGN
Human           SEYSSLRFKWFKNGNELNRKNKPQNIKIQKKPGKSELRINKASLADSGEYMCKVISKLGN
                *******************:*****:**********************************

Beta            DSASANITIVESNDLTTGMSASTERPYVSSESPIRISVSTEGANTSSSTSTSTTGTSHLI
Alpha           DSASANITIVESNDLTTGMSASTERPYVSSESPIRISVSTEGANTSSSTSTSTTGTSHLI
Human           DSASANITIVESNEIITGMPASTEGAYVSSESPIRISVSTEGANTSSSTSTSTTGTSHLV
                *************:: ***.**** .*********************************:

Beta            KCAEKEKTFCVNGGECFMVKDLSNPSRYLCKCPNEFTGDRCQNYVMASFYKHLGIEFMEA
Alpha           KCAEKEKTFCVNGGECFMVKDLSNPSRYLCKCQPGFTGARCTENVPMKVQTQE-----KA
Human           KCAEKEKTFCVNGGECFMVKDLSNPSRYLCKCQPGFTGARCTENVPMKVQNQE-----KA
                ********************************   *** ** : *  .. .:      :*

Beta            EELYQKRVLTITGICIALLVVGIMCVVAYCKTKKQRQKLHDRLRQSLRSERNNMVNIANG
Alpha           EELYQKRVLTITGICIALLVVGIMCVVAYCKTKKQRQKLHDRLRQSLRSERNNMVNIANG
Human           EELYQKRVLTITGICIALLVVGIMCVVAYCKTKKQRKKLHDRLRQSLRSERNNMMNIANG
                ************************************:*****************:*****

Beta            PHHPNPPPENVQLVNQYVSKNVISSEHIVEREVETSFSTSHYTSTAHHSTTVTQTPSHSW
Alpha           PHHPNPPPENVQLVNQYVSKNVISSEHIVEREVETSFSTSHYTSTAHHSTTVTQTPSHSW
Human           PHHPNPPPENVQLVNQYVSKNVISSEHIVEREAETSFSTSHYTSTAHHSTTVTQTPSHSW
                ********************************.***************************

Beta            SNGHTESIISESHSVIMMSSVENSRHSSPAGGPRGRLHGLGGPRECNSFLRHARETPDSY
Alpha           SNGHTESIISESHSVIMMSSVENSRHSSPAGGPRGRLHGLGGPRECNSFLRHARETPDSY
Human           SNGHTESILSESHSVIVMSSVENSRHSSPTGGPRGRLNGTGGPRECNSFLRHARETPDSY
                ********:*******:************:********:* *******************

Beta            RDSPHSERYVSAMTTPARMSPVDFHTPSSPKSPPSEMSPPVSSMTVSMPSVAVSPFVEEE
Alpha           RDSPHSERYVSAMTTPARMSPVDFHTPSSPKSPPSEMSPPVSSMTVSMPSVAVSPFVEEE
Human           RDSPHSERYVSAMTTPARMSPVDFHTPSSPKSPPSEMSPPVSSMTVSMPSMAVSPFMEEE
                **************************************************:*****:***
Beta            RPLLLVTPPRLREKKYDHHPQQLNSFHHNPAHQSTSLPPSPLRIVEDEEYETTQEYEPIQ
Alpha           RPLLLVTPPRLREKKYDHHPQQLNSFHHNPAHQSTSLPPSPLRIVEDEEYETTQEYEPIQ
Human           RPLLLVTPPRLREKKFDHHPQQFSSFHHNPAHDSNSLPASPLRIVEDEEYETTQEYEPAQ
                ***************:******:.********:*.***.******************* *

Beta            EPIKKVTNSRRAKRTKPNGHIANRLEMDSNPSSVSSNSESETEDERVGEDTPFLGIQNPL
Alpha           EPIKKVTNSRRAKRTKPNGHIANRLEMDSNPSSVSSNSESETEDERVGEDTPFLGIQNPL
Human           EPVKKLANSRRAKRTKPNGHIANRLEVDSNTSSQSSNSESETEDERVGEDTPFLGIQNPL
                **:**::*******************:***.** **************************

Beta            AASLEVAPAFRLAESRTNPAGRFSTQEELQARLSSVIANQDPIAV*
Alpha           AASLEVAPAFRLAESRTNPAGRFSTQEELQARLSSVIANQDPIAV*
Human           AASLEATPAFRLADSRTNPAGRFSTQEEIQARLSSVIANQDPIAV*
                *****.:******:**************:****************
```

Beta: mouse Type I NRG1β1 (AY648976.1：NCBI)
Alpha: mouse Type I NRG1α
Human: human Type INRG1α (NM_013964.3：NCBI)
EGF-like domain
α type or β1 type

Supplementary Figure S1.

Alignment of amino acid sequences of mouse Type I NRG1β1, mouse Type I NRG1α, and human Type I NRG1α. Amino acid homology are indicated by asterisks.

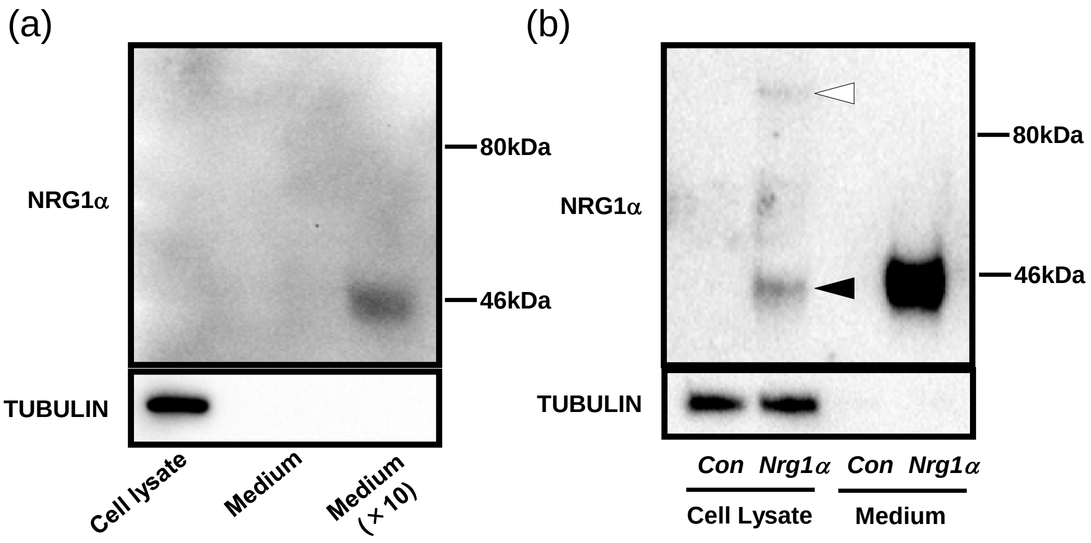

Supplementary Figure S2. Type I NRG1α secretes its ectodomain extracellularly from hepatocytes.

(a) Representative images of secreted Type I NRG1α protein from hepatocytes isolated from normal mouse liver. In the right lane (Medium x 10), conditioned medium was concentrated tenfold and then subjected to the analysis. Detection was performed using an anti-NRG1α antibody.

(b) Representative image of Type I NRG1α protein expression in whole cell lysate and conditioned medium of hepatocytes overexpressing *Nrg1α* gene. Detection was performed using an anti-NRG1α antibody. Black and white arrow head indicate a cleaved and uncleaved (full-length) Type I NRG1α protein, respectively.

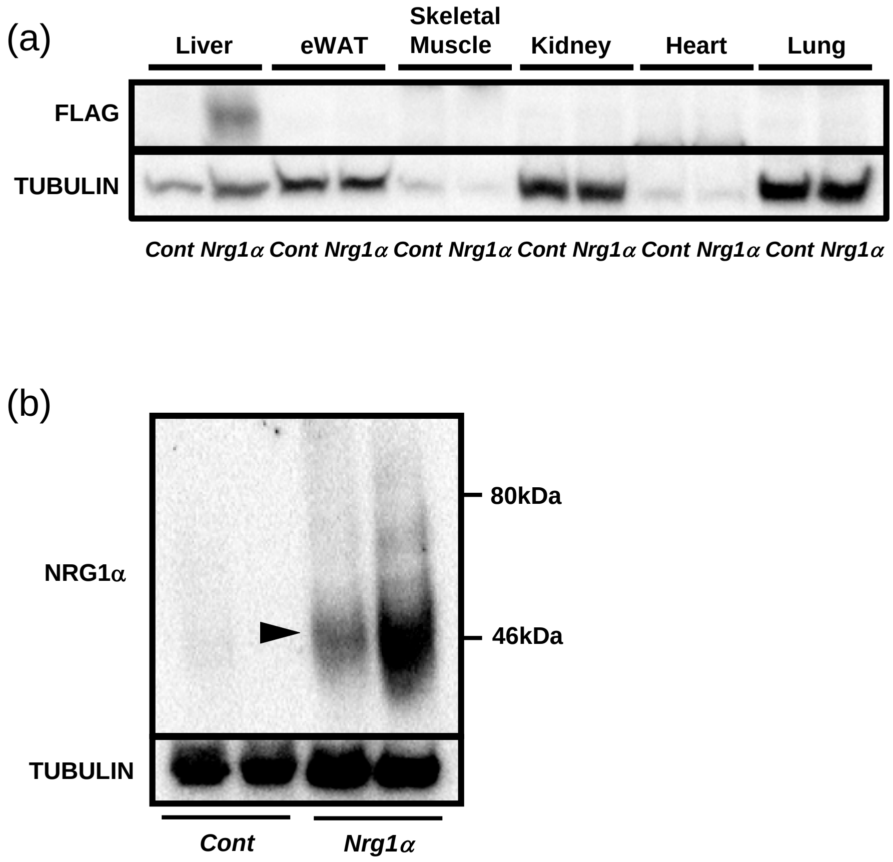

Supplementary Figure S3. Hydrodynamic tail vein injection allows *Type I Nrg1α* gene to be expressed exclusively in liver.

Representative image of Type I NRG1α protein expression in various organs (a) and liver (b) of mice injected with *Type I Nrg1α* tagged with a C-terminal Flag peptide-expressing plasmid. Empty vector was used as a control. Detection was performed using an anti-Flag antibody (a) and anti-NRG1α antibody (b), respectively. Black arrow head indicates the cleaved form of Type I NRG1α protein.

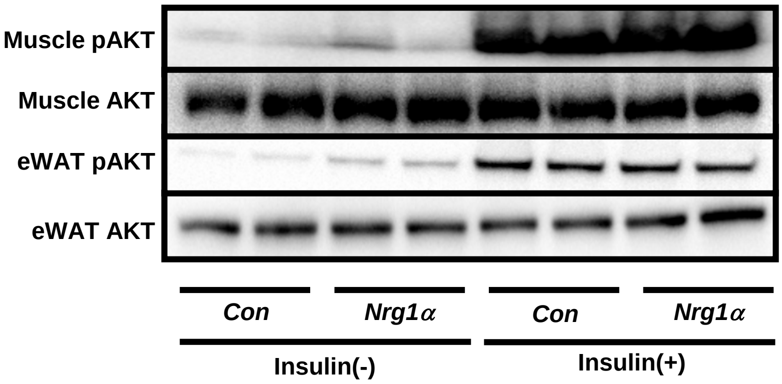

Supplementary Figure S4. ***Type I Nrg1α*** **overexpression in liver did not change insulin-induced AKT phosphorylation in peripheral tissues.**

Representative image of AKT phosphorylation in skeletal muscle and epididymal adipose tissue of mice overexpressing *Type I Nrg1α* in the liver.

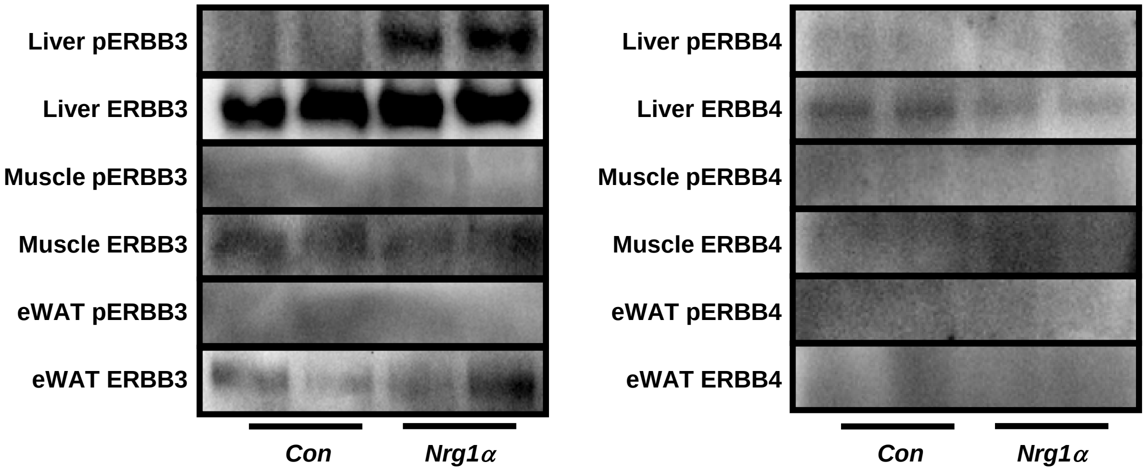

Supplementary Figure S5. Hepatic *Type I Nrg1α* overexpression selectively induced ERBB3 phosphorylation in the liver.

Representative image of ERBB3 and ERBB4 phosphorylation in liver, skeletal muscle, and epididymal adipose tissue of mice overexpressing *Type I Nrg1α* in the liver.

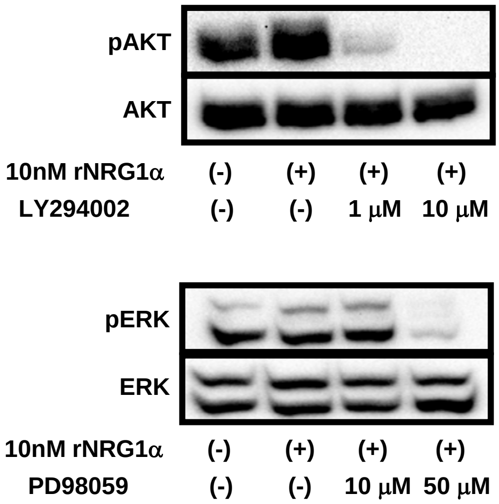

Supplementary Figure S6. Inhibitor for PI3K and ERK reduces phosphorylation of AKT and ERK in hepatocytes treated with rNRG1α.

Representative image of phosphorylation levels of AKT and ERK in hepatocytes treated with rNRG1α and ERBB signalling cascade inhibitors

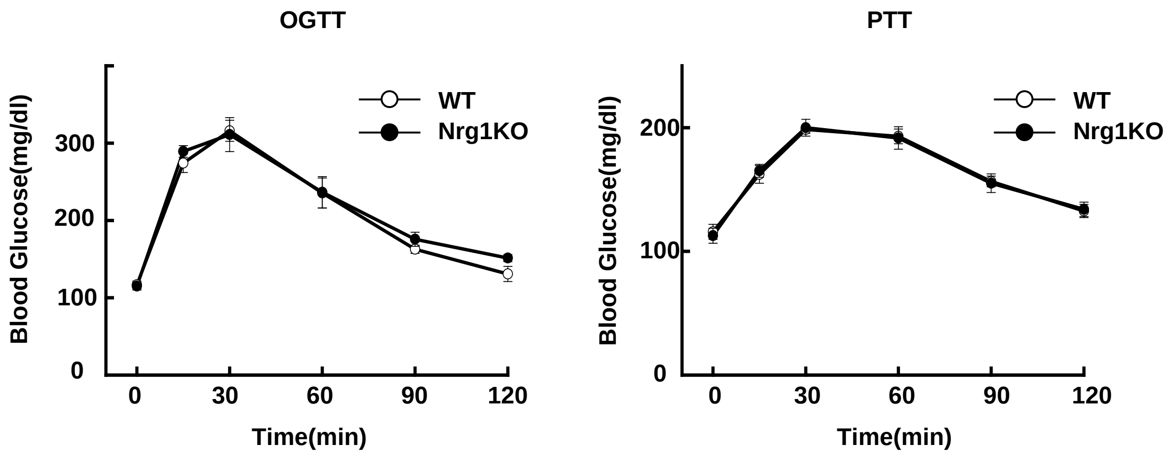

Supplementary Figure S7. A liver-specific deletion of *Nrg1* gene shows little effects on blood glucose levels in OGTT and PTT.

OGTT (left) and PTT (right) were performed using wild-type (WT) and liver-specific *Nrg1 gene* knockout (Nrg1KO) mice. n = 7 mice per group.

For PCR assay

Gene name	Forward primer	Reverse primer α	Reverse primer β
Type1-Nrg1	ATGTCTGAGCGCAAAGAAGGCAG	AGTACATCTTGCTCCAGTGAATCC	GGCCATTACGTAGTTTTGGCAA
Type2-Nrg1	ATGAGATGGCGACGCGCCCC		
Type3-Nrg1	TCCGCTGTTCTGGTCTCATC		
Type4-Nrg1	GGGCGAGTTCGCAGCACAG		
Type5-Nrg1	AGGCAAACCACCTACACCTACG		
Type6-Nrg1	CCCTTACTGTGGAGAGAAGC		

For qPCR assay

Gene name	Forward primer	Reverse primer	Probe
Pepck	TCCTGGCACCTCAGTGAAGAC	ACGTTGGTGAAGATGGTGTTTTT	CTGGCATTTGACTGGAACACGCCC
G6pase	GACCTGAGGAACGCCTTCTATG	GAGATTGATGCCCACAGTCTCTT	CTCTTTCCCATCTGGTTCCATC

Gene name	Forward primer	Reverse primer
Mcp1	AGCAACAGAGGGCGAAAGCTC	GGACTTCCGGGACTATCTCATG
Mcp2	GCCAGACCTGCAGAGAAACTC	ATAAACCCTGTAGCCATCAACAC
Erbb3	GAGTTACGGTGTAACCGTTTGG	GCTAACCGCTCTCCCTTCTC
Type1-Nrg1	AAGGGGAAGGGCAAGAAGAAG	TTCAATCTGGGAGGCAATGC
18S rRNA	GCTCGCTCCTCTCCTACTTG	CCCGTCGGCATGTATTAGCT

For siRNA assay

Gene name	Sequence
siNrg1	UGCAACUGGUGAAUCAAUAUGUAUCTA

Supplementary Table S1. Primer and probe sequences
